# The Different Responsiveness of C3- and C5-deficient Murine BM Cells to Oxidative Stress Explains Why C3 Deficiency, in Contrast to C5 Deficiency, Correlates with Better Pharmacological Mobilization and Engraftment of Hematopoietic Cells

**DOI:** 10.1007/s12015-024-10792-6

**Published:** 2024-09-28

**Authors:** Adrian Konopko, Agnieszka Łukomska, Magdalena Kucia, Mariusz Z. Ratajczak

**Affiliations:** 1 Center for Preclinical Studies and Technology, Laboratory of Regenerative Medicine at Medical University of Warsaw, Warsaw, Poland; 2 Stem Cell Institute at James Graham Brown Cancer Center, University of Louisville, 500 S. Floyd Street, Rm. 107, Louisville, KY 40202 USA

**Keywords:** C3 – deficient cells, C5- deficient cells, Oxidative stress, Complosome, Stem cell mobilization, Stem cell homing, Nlrp3 inflammasome

## Abstract

The liver-derived circulating in peripheral blood and intrinsic cell-expressed complement known as complosome orchestrate the trafficking of hematopoietic stem/progenitor cells (HSPCs) both during pharmacological mobilization and homing/engraftment after transplantation. Our previous research demonstrated that C3 deficient mice are easy mobilizers, and their HSPCs engraft properly in normal mice. In contrast, C5 deficiency correlates with poor mobilization and defects in HSPCs’ homing and engraftment. The trafficking of HSPCs during mobilization and homing/engraftment follows the sterile inflammation cues in the BM microenvironment caused by stress induced by pro-mobilizing drugs or myeloablative conditioning for transplantation. Therefore, to explain deficiencies in HSPC trafficking between C3-KO and C5-KO mice, we evaluated the responsiveness of C3 and C5 deficient cells to low oxidative stress. As reported, oxidative stress in BM is mediated by the activation of purinergic signaling, which is triggered by the elevated level of extracellular adenosine triphosphate (eATP) and by the activation of the complement cascade (ComC). In the current work, we noticed that BM lineage negative cells (lin^−^) isolated from C3-KO mice display several mitochondrial defects reflected by an impaired ability to adapt to oxidative stress. In contrast, C5-KO-derived BM cells show a high level of adaptation to this challenge. To support this data, C3-KO BM lin^−^ cells were highly responsive to eATP stimulation, which correlates with enhanced levels of reactive oxygen species (ROS) generation and more efficient activation of intracellular Nlrp3 inflammasome. We conclude that the enhanced sensitivity of C3-KO mice cells to oxidative stress and better activation of the Nox2-ROS-Nlrp3 inflammasome signaling axis explains the molecular level differences in trafficking between C3- and C5-deficient HSPCs.

**Graphical Abstract:**

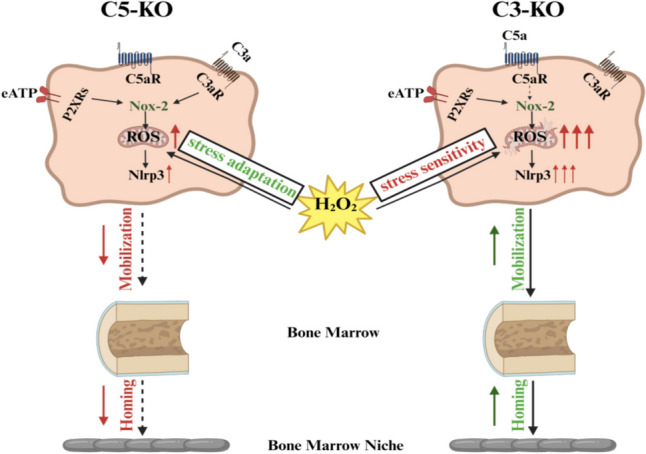

## Introduction

Our previous work has documented an important role of complement cascade (ComC) in the trafficking of hematopoietic stem/progenitor cells (HSPCs), as seen during pharmacological mobilization and homing/engraftment after transplantation to the myeloablated host [1, 2]. However, we noticed some differences in C3-KO and C5-KO mice [3–6]. Firstly, while C3-KO animals turned out to be easy mobilizers [3], C5-KO mice released poorly HSPCs from bone marrow (BM) into peripheral blood (PB) [4]. At that time, we interpreted this discrepancy as the luck of BM-expressing C3 cleavage fragments C3a and C3b that facilitate the retention of HSPCs in BM niches and their engraftment after transplantation [3]. We also stated that coordinated activation of both proximal (C3) and distal (C5) elements of ComC is required for optimal mobilization [1, 2], and postulated that both liver-derived and intrinsic complement expressed in the cells (complosome) play an essential role [7, 8].

Evidence has accumulated that the administration of pro-mobilizing drugs, such as cytokine-granulocyte-colony stimulating factor (G-CSF) or myeloablative conditioning for transplantation, is a driving force for the trafficking of HSPCs [9]. It is explained by the fact that administering a pro-mobilizing drug or myeloablative treatment induces the BM microenvironment state of sterile inflammation [2]. This state is initiated by releasing extracellular adenosine triphosphate (eATP) that activates purinergic signaling [10] and complement cascade (ComC) [2, 11]. Moreover, both these signaling pathways potentiate each other’s activity [1, 2]. Our most recent research also established the importance of eATP and ComC-cleavage fragments as activators of NADPH oxidase 2 (Nox2)—reactive oxygen species (ROS)—Nlrp3 inflammasome signaling axis required for the optimal trafficking of HSPCs [12].

Therefore, to address better differences in mobilization efficacy between C3-KO [3] and C5-KO [4] mice, we focused on the responsiveness of cells from these animals to the low oxidative stress, ROS generation, and activation of Nlrp3 inflammasome. Herein, we report that BM lineage negative cells (lin^−^) isolated from C3-KO mice display several mitochondrial defects reflected by an impaired ability to adapt to oxidative stress. In contrast, C5-KO BM lin^−^ cells show a higher level of resistance to environmental changes, including a low level of oxidative stress. We noticed that C3-KO BM lin^−^ cells were highly responsive to ATP stimulation that correlated with enhanced levels of reactive oxygen species (ROS) generation and better activation of intracellular Nlrp3 inflammasome. Based on this data, we conclude that enhanced sensitivity of C3-KO mice cells to oxidative stress and better activation of Nox2-ROS-Nlrp3 inflammasome signaling axis explains at molecular level differences in mobilization as well homing/engraftment efficacy between C3-KO and C5-KO mice.

## Material and Methods

### Animals

Pathogen-free, 6–8-week-old female C57BL/6 J wild-type (WT), C3-KO (B6.129S4-*C3*^*tm1Crr*^/J, strain#029661) and C5-KO (B10.D2-*Hc*^*0*^* H2*^*d*^* H2*-*T18*^*c*^/oSnJ, strain#000461) were purchased from the Jackson Laboratory (Bar Harbor, ME; USA) or the Central Laboratory for Experimental Animals, Medical University of Warsaw. Before obtaining bone marrow, mice were housed in the animal facility with a 12-h light/12-h dark cycle (lights on from 7:00 AM to 7:00 PM), with unlimited access to water and standard rodent food.

### Lineage-negative Cells Depletion

Lin^−^ -cells were purified from the WT C3-KO and C5-KO mice using a Direct Lineage Cell Depletion Kit (Miltenyi Biotec, Bergisch Gladbach, Germany). Briefly, cells were incubated for 10 min at 4 °C with MicroBeads conjugated to monoclonal antibodies against CD5, CD11b, CD45R (B220), Anti-Gr-1 (Ly-6G/C), 7–4, and Ter-119 (Direct Lineage Cell Depletion Kit, Miltenyi Biotec, Bergisch Gladbach, Germany). Following incubation, the cells were washed with 3 ml of phosphate-buffered saline (PBS) and centrifuged at 1800 rpm for 10 min at 4 °C. The cell pellet was resuspended in 1 ml of PBS, and the cell suspension was applied to an LC column (Miltenyi Biotec, Bergisch Gladbach, Germany) placed in a magnetic field (Manual Cell Separator, Miltenyi Biotec, Bergisch Gladbach, Germany) after rinsing the column with 3 ml of PBS. The column was then washed with 3 ml of PBS to elute and collect the purified Lin^−^ cells [12].

### Mitochondria Functionality Measurement—Oxygen Consumption Rate (OCR)

Bone marrow stem cells were isolated from WT, C3-KO, and C5-KO mice. Lin^−^ -cells were then purified using an LC column as previously described. The cells were then treated with 10 µM H_2_O_2_ for 15 min and washed with phenol-red-free RPMI media. Following the manufacturer’s protocol, the oxygen consumption rate was measured at 37 °C using a Seahorse XF HS MINI (Agilent Technologies, Santa Clara, CA, United States) with the Cell Mito Stress Test Kit. Briefly, 400,000 cells per well in 180 μl of phenol-red-free RPMI media were added to a Seahorse XFp PDL miniplate and incubated at 37 °C without CO_2_ for 1 h. OCR measurements were taken from a 90% confluent monolayer culture. A protocol was followed to assess mitochondrial function indices, involving sequential injections of oligomycin, FCCP, and a mixture of rotenone and antimycin A through the ports of Seahorse Flux Pak cartridges. The final concentrations of these compounds were 1.5 μM, 2 μM, and 0.5 μM, respectively, enabling the determination of basal, maximal respiratory, spare respiratory capacity, and ATP production. These parameters were calculated, including non-mitochondrial oxygen consumption correction, using the following formulas:

$$\begin{array}{c}\mathrm{Basal}\;\mathrm{Respiratory}\;={\mathrm{OCR}}_{\mathrm{initial}}-{\mathrm{OCR}}_{\mathrm{RO}/\mathrm{AA}}\\\mathrm{Maximum}\;\mathrm{Respiratory}\;={\mathrm{OCR}}_{\mathrm{FCCP}}-{\mathrm{OCR}}_{\mathrm{RO}/\mathrm{AA}}\\\mathrm{Spare}\;\mathrm{Respiratory}\;\mathrm{Capacity}\;={\mathrm{OCR}}_{\mathrm{FCCP}}-{\mathrm{OCR}}_{\mathrm{initial}}\\\mathrm{ATP}\;\mathrm{production}\;={\mathrm{OCR}}_{\mathrm{initial}}-{\mathrm{OCR}}_{\mathrm{oligomycin}}\end{array}$$

### Reactive Oxygen Species-level Measurements

The CM-H_2_DCFDA (chloromethyl derivative of 2’,7'-dichlorodihydrofluorescein diacetate) (Thermo Fisher Scientific, Cat. No. C6827) was used to measure ROS levels, following the standard procedure. Briefly, Lin^−^ cells (10 000 per 100 µl media) were pre-treated with eATP (1 nM and 10 µM, Merck, Darmstadt, Germany), eATP (10 μM) + Mito-TEMPO (10 μM, Santa Cruz Biotechnology, TX, USA), C3a (1 nM and 10 nM, Bio-Techne, MN, USA), C3a (10 nM) + Mito-TEMPO (10 μM) and C5a (1 nM and 10 nM, Bio-Techne, MN, USA), C5a (10 nM) Mito-TEMPO (10 μM) for 16 h. The cells were then centrifuged at 1800 rpm for 10 min, and the resulting cell pellets were resuspended in 1 ml of phenol-red free RPMI medium with CM-H_2_DCFDA (final concentration 5 µM) and incubated at 37 °C for 30 min. Subsequently, the cells were centrifuged (1800 rpm) for 10 min and suspended in 200 µl of phenol-red free RPMI medium. The fluorescence of cells was recorded at λ_ex_ = 488 nm and λ_em_ = 523 nm using SpectraMax iD3 Multi-Mode Microplate Readers (Molecular Devices, CA, USA). ROS levels were quantified using CM-DCF fluorescence intensity and expressed as a percentage of the control (non-treated cells).

### Nlrp3 Inflammasome Activation Measurements

The activity of caspase-1 in Lin^−^ cells isolated from WT, C3-KO, and C5-KO BM was measured using the Caspase-Glo® 1 Inflammasome Assay (Promega, USA), following the manufacturer’s protocols. Control samples (non-treated cells) and cells treated with eATP (10 μM), eATP (10 μM) + Mito-TEMPO (10 μM), C3a (10 nM), C3a + Mito-TEMPO (10 μM), C5a (10 nM) and C5a + Mito-TEMPO (10 μM) were incubated for 3 h at 37 °C in 5% CO_2_. Subsequently, the cells were harvested, and 50,000 cells were plated in 96-well plates. Caspase-Glo® 1 Reagent or Caspase-Glo® 1 YVAD-CHO Reagent was added (100 μl/well), gently mixed, and incubated at room temperature. Luminescence was recorded at 60, 90, and 120 min [13] using a SpectraMax iD3 Multi-Mode Microplate Readers (Molecular Devices, CA, USA).

### Statistical Analysis

All results are expressed as the mean ± SD from at least three independent experiments. Statistical analyses were conducted using GraphPad Prism 9.0 (GraphPad Software Inc., La Jolla, CA, USA). Data was analyzed using multiple unpaired t-tests, with significance set at *p* ≤ 0.05. Statistical significance is as follows: * *p* < 0.05, ** *p* < 0.01, *** *p* < 0.001, **** *p* < 0.0001.

## Results

### C3-KO Mice, Compared to C5-KO Mice, Display Distinct Mitochondrial Adaptive Responsiveness to Mild Oxidative Stress

Cells isolated from WT, C3-KO, and C5-KO were evaluated for their redox characteristics during steady-state conditions and after exposure to modifiers of mitochondrial function. Accordingly, we measured oxygen consumption (OCR) during steady-state conditions and after exposure to mitochondrial ATP synthase inhibitor oligomycin, followed by maximal stimulation of respiratory rate induced by the uncoupler carbonyl cyanide-4-(trifluoromethoxy) phenylhydrazone (FCCP), and finally after exposure of cells to Rotenone/Actinomycin A that inhibits mitochondrial respiratory chain complex I and III, thereby entirely blocking the electron transport chain [14]. This allowed us to measure crucial parameters in WT, C3a-KO, and C5-KO cells, describing mitochondrial adaptive responsiveness to stress.

**Fig. 1:**
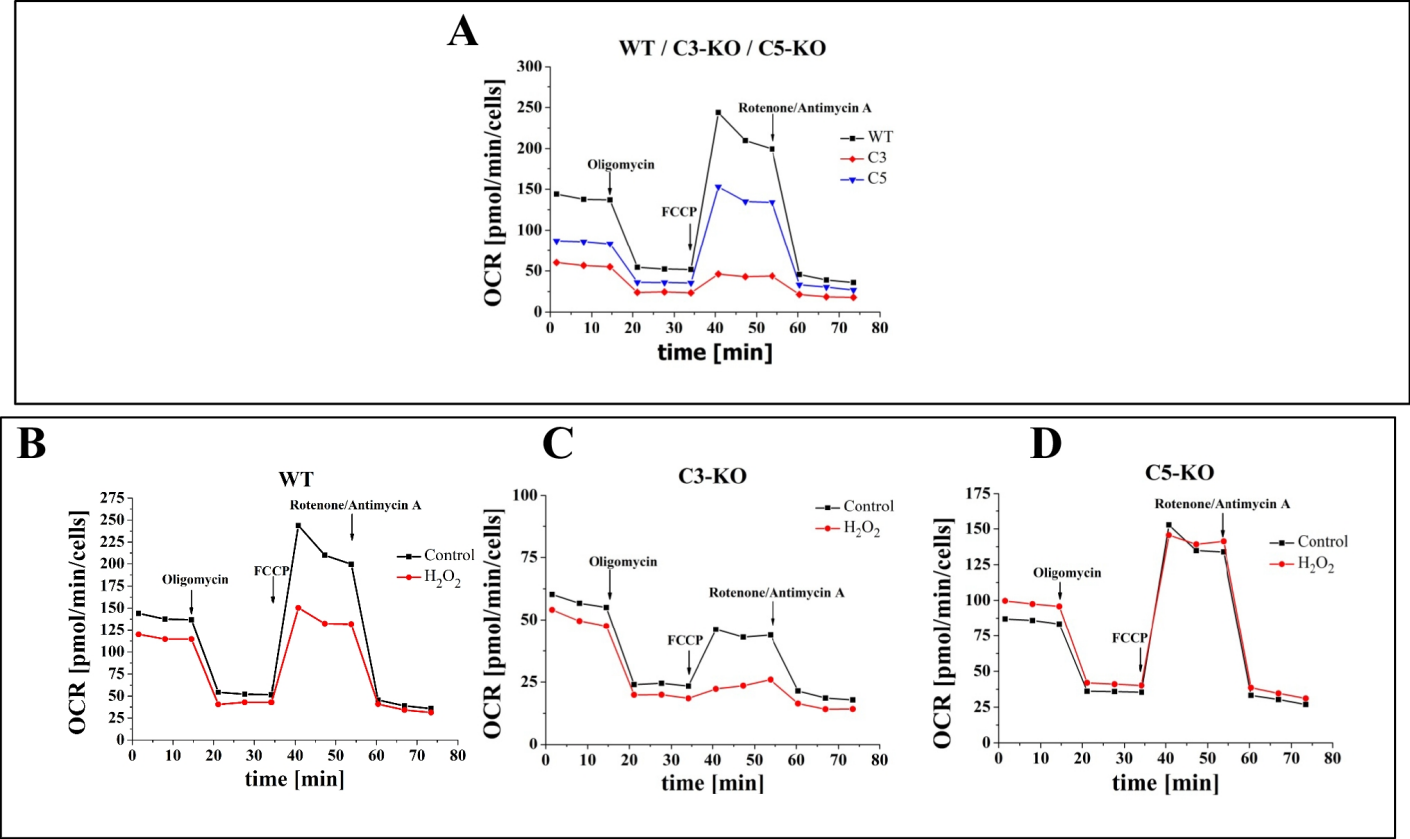
Mitochondrial Functionality Assessment—Oxygen consumption rate (OCR). Mitochondrial activity of Lin^−^ cells isolated from BM of WT, C3-KO, and C5-KO mice exposed to 10 µM H_2_O_2_. **A**) Panel A compares OCR in cells isolated from WT, C3-KO, and C5-KO mice. **B-D**) Panels B-D compare OCR in non-treated cells and treated with 10 µM H_2_O_2_ (**B** – WT, **C** – C3-KO and **D** – C5-KO). Values are expressed as the mean ± standard deviation (SD) from at least three independent experiments (*n* = 3). Statistical analysis was performed using unpaired t-tests. Statistical significance is indicated by *p ≤ 0.05, **p ≤ 0.01, ***p ≤ 0.001

**Fig. 2:**
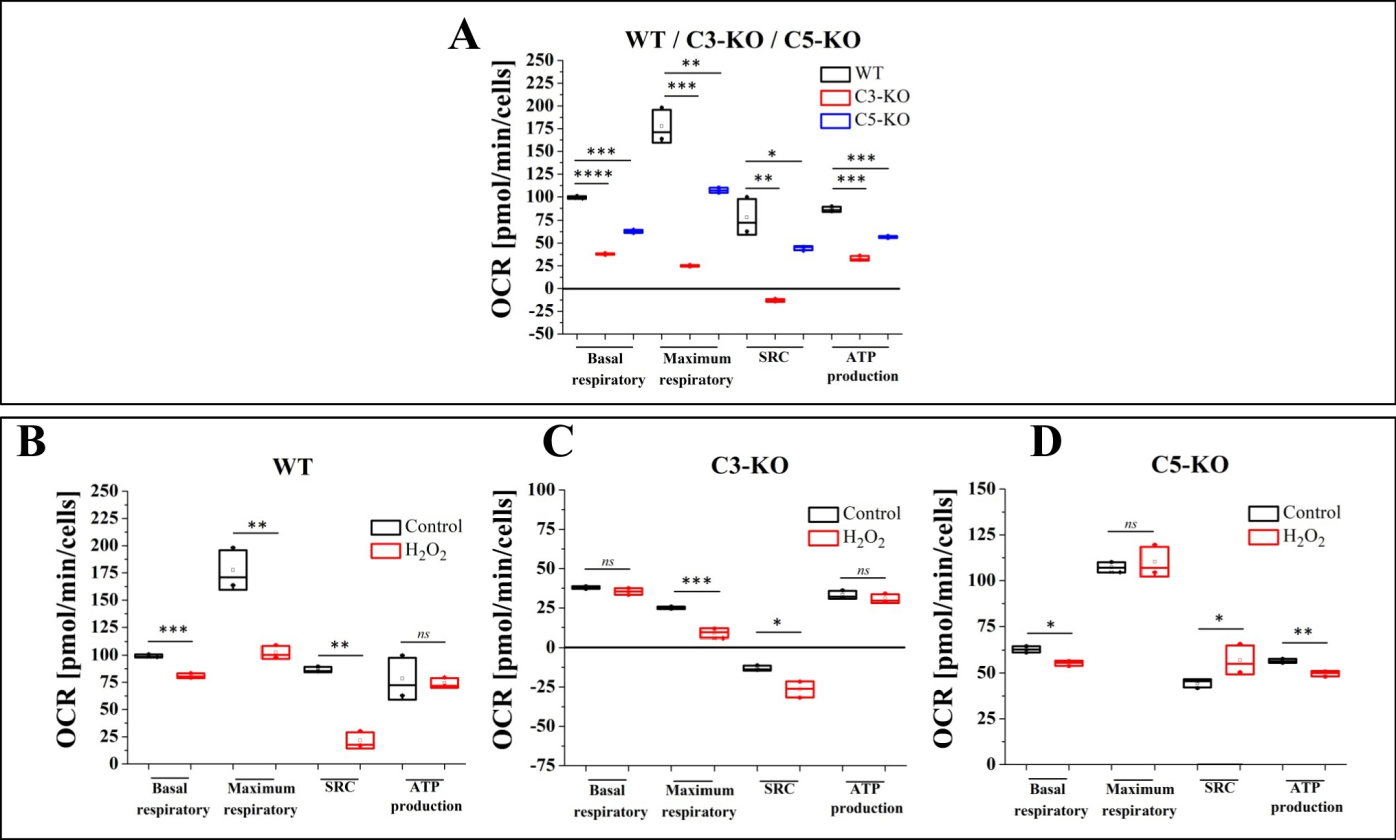
Mitochondrial Functionality Assessment. Panel **A** compares basal respiration, maximum respiration, spare respiratory capacity (SRC), and ATP production between cells isolated from WT, C3-KO, and C5-KO under steady-state conditions. Panels **B-D** Show mitochondrial parameters in cells treated with 10 µM H_2_O_2_ compared to untreated cells: **B** – WT; **C** – C3-KO; **D** – C5-KO. Values are expressed as the mean ± standard deviation (SD) from at least three independent experiments (*n* = 3). Statistical analysis was performed using unpaired t-tests. Statistical significance is indicated by *p ≤ 0.05, **p ≤ 0.01, ***p ≤ 0.001

Lin^−^ BM stem cells isolated from C3-KO and C5-KO under steady-state conditions exhibited significantly lower basal and maximal respiratory levels (Figs. 1A and 2A), indicating a compromised mitochondrial function in these mutant mice. At the same time, C3-KO mice, in contrast to C5-KO mice, demonstrated a complete inability to adapt to oxidative stress, as evidenced by a negative Spare Respiratory Capacity (SRC < 0) Fig. 2A. In contrast, C5-KO mice retained some capacity to manage oxidative stress, as indicated by a positive SRC (SRC > 0). These findings highlight distinct differences in mitochondrial function between these two complement-deficient cells.

To further assess mitochondrial functionality, we evaluated the impact of mild oxidative stress (10 µM H_2_O_2_) (Figs. 1B–D and 2B–D). Our observations revealed that the SRC parameter increased in C5-KO mice during oxidative stress (Fig. 2D), suggesting an enhanced ability to adapt to environmental changes. In contrast, lin^−^ cells isolated from WT mice significantly decreased SRC (Fig. 2B), indicating a lower adaptive capacity than C5-KO animals. Moreover, the decrease in SRC observed in C3-KO cells following H_2_O_2_ treatment (Fig. 2C) confirmed a complete lack of adaptive response to oxidative stress, consistent with the results observed under steady-state conditions.

### Effect of C3a, C5a, and Eatp on ROS Production

**Fig. 3:**
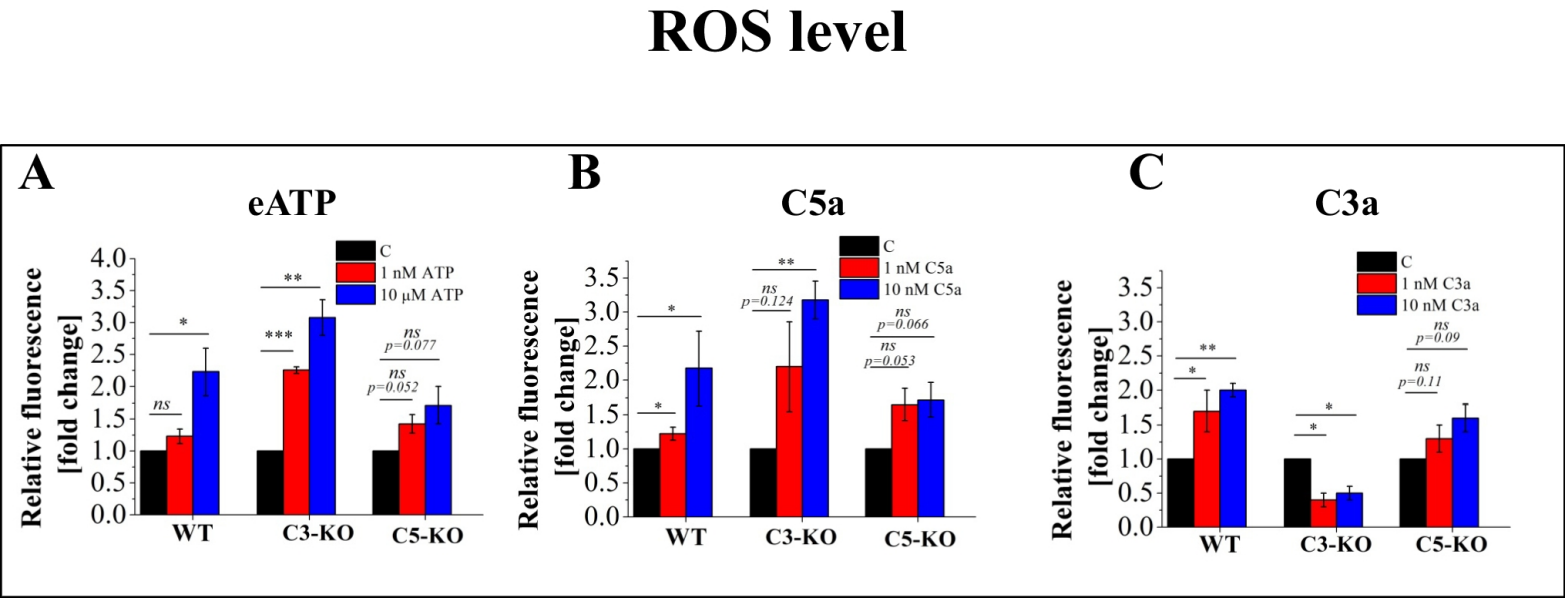
Analysis of the ROS level in BM Lin^−^ stem cells isolated from WT, C3-KO, and C5-KO mice following treatment with eATP (1 nM and 10 µM), C3a (1 nM and 10 nM) and C5a (1 nM and 10 nM) for 16 h. Panel **A** shows relative fluorescence following treatment with eATP. Panel **B** shows relative fluorescence following treatment with C5a. Panel **C** shows relative fluorescence following treatment with C3a. All results are expressed as a percentage of the control (non-treated cells). Values are expressed as the mean ± standard deviation (SD) from at least three independent experiments (*n* = 3). Statistical analysis was performed using unpaired t-tests. Statistical significance is indicated by *p ≤ 0.05, **p ≤ 0.01, ***p ≤ 0.001

Based on data that the stimulation of stem cells with C3a, C5a, and eATP is known to influence HSPCs mobilization [15] as well as homing, and engraftment [16], we assessed the ROS levels in lin^−^ cells from WT, C3-KO, and C5-KO mice following treatment with these stimulators (Fig. 3). Our analysis revealed a significant increase in ROS production in cells isolated from WT mice upon exposure to all these compounds. In contrast, cells from C5-KO mice exhibited only a slight, non-significant increase in ROS levels after exposure, supporting the notion that C5-KO mice have a slightly enhanced capacity to adapt to environmental changes. Conversely, C3-KO cells displayed heightened sensitivity to eATP and C5a, with ROS levels surpassing those observed in WT cells. Interestingly, at the same time, treatment with C3a produced the opposite effect in C3-KO cells, resulting in a statistically significant two-fold decrease in ROS levels.

### Effect of C3a, C5a and Eatp on Nlrp3 Activation

**Fig. 4:**
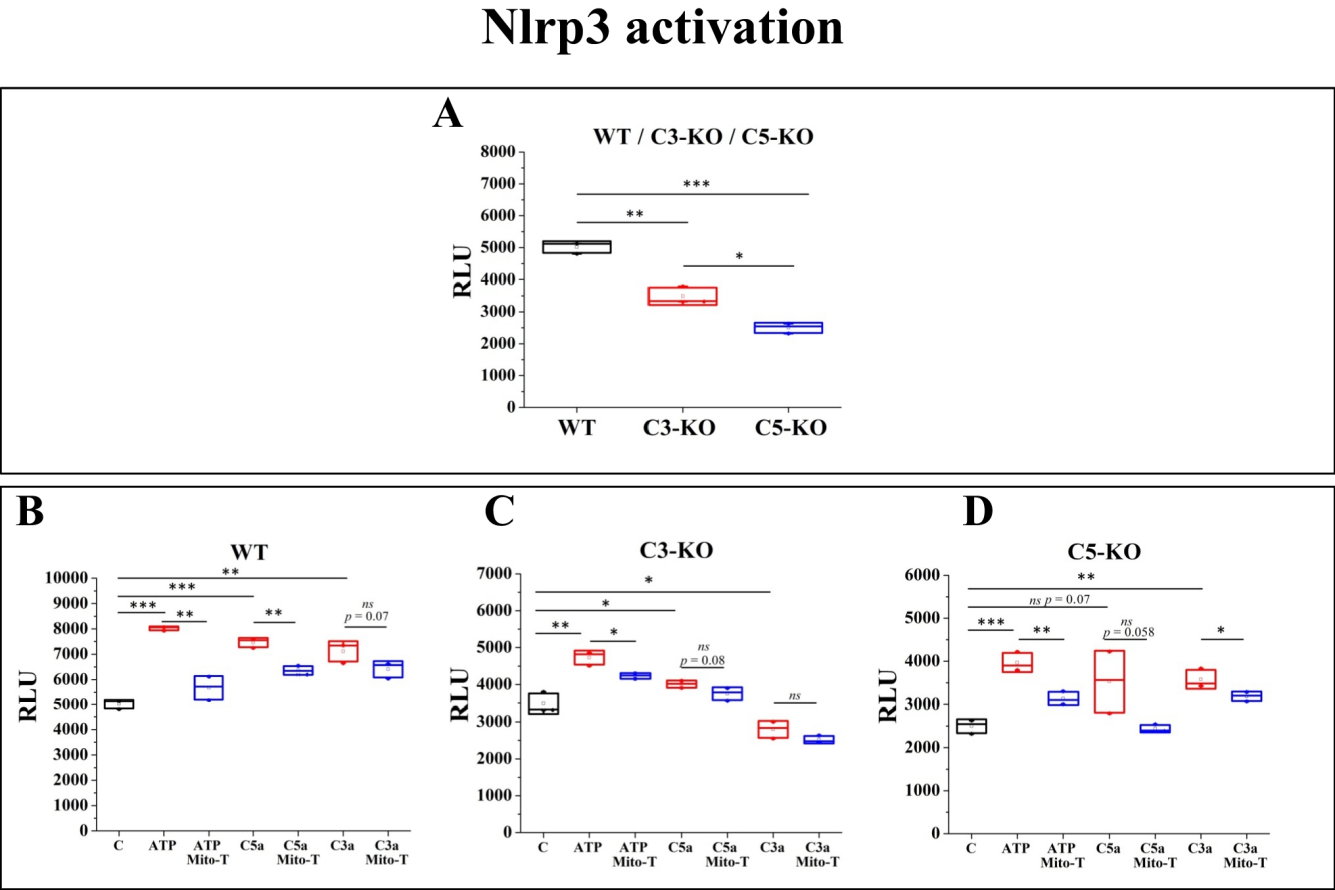
Nlrp3 inflammasome activation measurements. Panel **A** Nlrp3 inflammasome activation was measured in Lin^−^ BM stem cells isolated from WT, C3-KO, and C5-KO under steady-state conditions. Panels **B-D** Analysis of Nlrp3 inflammasome activation following treatment with eATP (10 μM), eATP (10 μM) + Mito-TEMPO (10 μM), C3a (10 nM), C3a + Mito-TEMPO (10 μM), C5a (10 nM) and C5a + Mito-TEMPO (10 μM) for 3 h. Panel **B** – WT, Panel **C** – C3-KO, Panel **D** – C5-KO. Values are expressed as the mean ± standard deviation (SD) from at least three independent experiments (*n* = 3). Statistical analysis was performed using unpaired t-tests. Statistical significance is indicated by *p ≤ 0.05, **p ≤ 0.01, ***p ≤ 0.001

Our previous work postulated that ROS levels are closely associated with Nlrp3 inflammasome activation through the Nox2-ROS-Nlrp3 signaling axis [12]. We analyzed Nlrp3 activation in lin^−^ BM stem cells isolated from WT, C3-KO, and C5-KO mice to gain deeper insight into this molecular mechanism. Under steady-state conditions, we observed that Nlrp3 activation was lower in cells from both mutant mice than in WT, with the lowest activation in C5-KO cells (Fig. 4A).

We then assessed the effects of C3a, C5a, and eATP on Nlrp3 activation (Fig. 4B-D). In WT cells, treatment with these activators significantly increased Nlrp3 activation (Fig. 4B). In C3-KO cells, eATP and C5a triggered heightened Nlrp3 activation as compared to C5-KO cells, consistent with the increased ROS production observed following treatment with these activators, whereas C3a had no effect. Conversely, in C5-KO cells, Nlrp3 activation increased following eATP and C3a treatment, while C5a caused only a slight, non-significant increase.

To better understand the molecular basis of these effects, we combined the activator treatments with Mito-TEMPO, a well-known mitochondrial antioxidant. We found that, in all experimental conditions, treatment with eATP, C5a, C3a, and Mito-TEMPO led to a decrease in Nlrp3 activation (blue boxes in Fig. 4B–D). These findings further support the role of ROS production in Nlrp3 inflammasome activation in response to eATP, C3a, and C5a.

### The Mitochondrial Antioxidant Mito-TEMPO Inhibits ROS Production Induced by eATP, C3a, and C5a

**Fig. 5:**
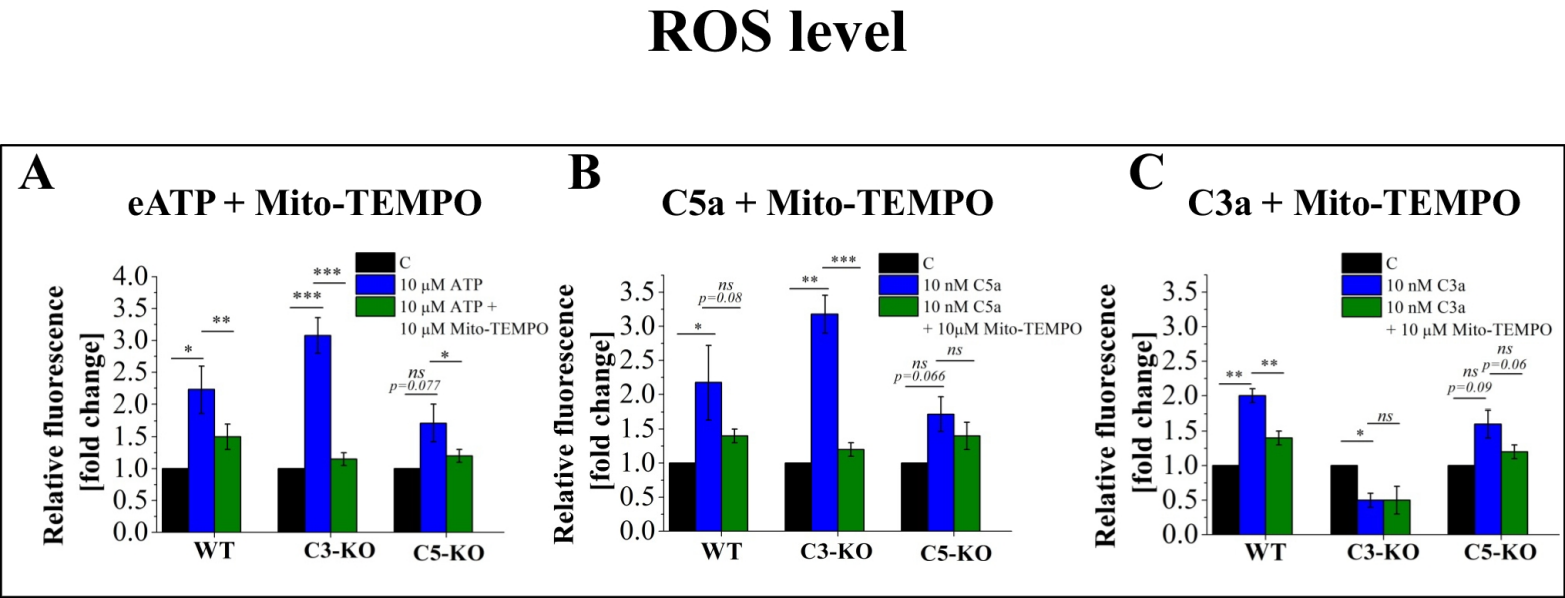
Analysis of the ROS inhibition by mitochondrial antioxidant Mito-TEMPO in BM Lin^−^ stem cells isolated from WT, C3-KO and C5-KO mice following treatment with eATP (10 µM), eATP (10 µM) + Mito-TEMPO (10 µM), C3a (10 nM), C3a (10 nM) + Mito-TEMPO (10 µM) and C5a (10 nM), C5a + Mito-TEMPO (10 µM) for 16 h. Panel **A** shows relative fluorescence following treatment with eATP and eATP + Mito-TEMPO. Panel **B** shows relative fluorescence following treatment with C5a and C5a + Mito-TEMPO. Panel **C** shows relative fluorescence following treatment with C3a and C3a + Mito-TEMPO. All results are expressed as a percentage of the control (non-treated cells). Values are expressed as the mean ± standard deviation (SD) from at least three independent experiments (*n* = 3). Statistical analysis was performed using unpaired t-tests. Statistical significance is indicated by *p ≤ 0.05, **p ≤ 0.01, ***p ≤ 0.001

Given that treatment with activators and Mito-TEMPO reduces Nlrp3 inflammasome activation, we sought to corroborate these findings by assessing ROS production. Specifically, we investigated whether treatment with eATP, C3a, and C5a, combined with Mito-TEMPO, would decrease cell ROS levels (Fig. 5). Our results confirmed that Mito-TEMPO consistently reduced ROS production in all cases where activator treatments had previously induced ROS. Notably, the most significant reduction was observed in cells isolated from C3-KO mice, aligning with our earlier findings that these cells are susceptible to oxidative stress.

## Discussion

We demonstrated in the past that while C3 deficient mice are easy mobilizers and their HSPCs engraft properly in normal mice [3, 5], C5 deficient mice mobilized poorly and showed defects in HSPCs homing/engraftment after transplantation into normal recipients [4, 6]. As a follow-up, the seminal observation of our current work is evidence that enhanced sensitivity of C3-KO mice cells to oxidative stress and better activation of Nox2-ROS-Nlrp3 inflammasome signaling axis explains at molecular level differences in HSPCs trafficking between C3-KO and C5-KO mutant mice.

The HSPCs are retained in the BM microenvironment in specific stem cell niches [17]. They could be released into PB upon exposure to pro-mobilizing agents such as cytokine granulocyte colony-stimulating factor (G-CSF) or CXCR4 receptor antagonist Plerixafor [18]. Subsequently, these cells are isolated from the PB by leukapheresis and are employed as a source of cells for hematopoietic graft [19]. Hematopoietic transplantation itself relies on intravenous infusion of harvested hematopoietic cells that navigate from the PB stream into the BM microenvironment [20]. Both pharmacological mobilization of the cell donor and myeloablative conditioning of the recipient before transplantation induce in the BM state of sterile inflammation [2] triggered by ComC and purinergic signaling [15, 16]. In addition, evidence accumulated that not only circulating in PB liver-derived ComC fragments such as C3a and C5a orchestrate the trafficking of HSPCs, but also ComC elements expressed intracellularly as complosome in hematopoietic cells and BM microenvironment cells play an important role [6]. Of note is that C5aR receptors have been identified as being expressed in mitochondria [7, 8]. Novel evidence also indicates that eATP, a key element in purinergic signaling released from the cells as a trigger of sterile inflammation, may also affect HSPCs in an autocrine manner by activating purinergic receptor P2X7 expressed on cell mitochondria [21].

Sterile inflammation leads to the activation of Nox2 co-expressed in a functional complex with i) several signaling receptors on the cell surface and ii) is present directly in mitochondria and generates in cytosol ROS [22]. The most prominent ROS is hydrogen peroxide (H_2_O_2_) [23], employed in our studies to mimic the effect of oxidative stress in experimental *in vitro* models. ROS is well known as a potent activator of intracellular pattern recognition receptor – Nlrp3 inflammasome that regulates migration and metabolism of HSPCs [24]. We have presented in the past data that the Nox2-ROS-Nlrp3 inflammasome signaling axis expressed both in HSPCs as well as in cells in the hematopoietic microenvironment is crucial for optimal mobilization and homing/engraftment of HSPCs [12]. This explains why we focused on the activity of this axis in our current experiments.

To explain the difference in trafficking of HSPCs between C3a-KO and C5-KO mice, we demonstrate herein that under steady-state conditions, lin^−^ BM cells isolated from C3-KO and C5-KO exhibited significantly lower basal and maximal respiratory levels, indicating a compromised mitochondrial function. This defect was more prominent in C3-KO cells, demonstrating a complete inability to adapt to oxidative stress, as evidenced by a negative SRC [25]. This exciting observation requires further studies to identify the exact mechanism responsible for this mitochondria deficiency observed in C3-deficient mice.

Since mitochondria are an important source of ROS as a crucial activator of Nlrp3 inflammasome [22, 24, 26], we evaluated the impact of mild oxidative stress in response to H_2_O_2_ on the respiratory functions of these organelles. The decrease in SRC in C3-KO cells following H_2_O_2_ treatment revealed a complete lack of adaptive response to oxidative stress, consistent with the results observed under steady-state conditions. In contrast, increased SRC parameters in cells from C5-KO mice indicate an enhanced ability to adapt to environmental changes. The lack of adaptive response of C3-KO cells to oxidative stress confirms that these cells cannot adapt to oxidative stress and, thus, are highly susceptible to this challenge, as seen, for example, during sterile inflammation. The increased sensitivity of C3-KO cells to oxidative stress, as compared to C5-KO cells, was demonstrated by their enhanced sensitivity to eATP and C5a stimulation, leading to an increase in ROS level, surpassing even those observed in WT cells.

As mentioned above, ROS is a potent stimulator of the Nlrp3 inflammasome that orchestrates the trafficking of HSPCs [12, 25, 27]. Under steady-state conditions, Nlrp3 activation was lower in cells in both mutant mice. Nevertheless, this basic activation was higher in C3-deficient cells than in C5-KO ones. Moreover, upon stimulation by eATP and C5a, Nlrp3 inflammasome activation was triggered at a higher level in C3-KO cells. Interestingly, we also noticed that the exposure of cells to mitochondrial antioxidant Mito-TEMPO [28] only partially inhibited Nlrp3 inflammasome activation. This suggests a role ROS-derived from other non-mitochondrial sources that partially contributed to this effect.

We know that the lin^−^ BM cell population is enriched for HSPCs and some other cells that constitute the hematopoietic BM microenvironment [27, 29]. Therefore, further studies on the more purified population of HSPCs and BM non-hematopoietic cells will be needed. To support this role of the hematopoietic microenvironment, we reported in the past that both C3-KO [5] and C5-KO [6] show microenvironmental defects in homing and engraftment of transplanted normal BM cells. Moreover, the observed differences in responsiveness of C3- and C5-deficient cells to oxidative stress are also essential to better understanding the biological effects of intracellular complement (complosome) [7, 8].

In conclusion, the enhanced sensitivity of C3-KO mice cells to oxidative stress and better activation of the Nox2-ROS-Nlrp3 inflammasome signaling axis in cells from this mutant animals explains differences in mobilization and homing/engraftment efficacy reported between C3-KO [3, 5] and C5-KO [4, 6] murine strains.

## Data Availability

Detailed data is available upon request.
